# The Principles and Procedures of Ultrasound-guided Anesthesia Techniques

**DOI:** 10.7759/cureus.2980

**Published:** 2018-07-13

**Authors:** Jeffrey Huang, Jinlei Li, Hong Wang

**Affiliations:** 1 Anesthesiology, University of Central Florida, Orlando, USA; 2 Anesthesiology, Yale University, New Haven , USA; 3 Anesthesiology, West Virginia University, Morgantown, USA

**Keywords:** inexperienced users, ultrasound, techniques, regional anesthesia

## Abstract

For inexperienced users, training with phantoms is an important part of training. Inexperienced users can teach themselves to gain significant procedural skills. Participating in training courses or practising with experts can enhance the outcomes. Inexperienced users need to understand the indications, clinical pearls, and pitfalls of each procedure to avoid potential complications. Inexperienced users can also train and teach themselves to become proficient in ultrasound techniques.

## Introduction and background

In 1978, La Grange and colleagues reported the first use of ultrasounds for nerve blocks [1]. Accumulating evidence quickly demonstrated that ultrasound-guided nerve blocks offer improved block quality, shorter performance times, and lower complication rates [2-3]. The proper application of ultrasound-guided nerve blocks requires ultrasound equipment, a provider’s understanding of sonoanatomy, knowledge of ultrasound physics, and skills in needle and nerve structures visualization [3]. We here present the essential training information for inexperienced providers who wish to become proficient in doing ultrasound-guided nerve blocks.

## Review

Basic ultrasound knowledge

Ultrasound is a high-frequency sound wave that generates images by receiving the signals reflected back by the interface of the tissues with different acoustic impedances. The tissue acoustic impedance is dependent on the property of the tissue such as density. The lung mainly contains air and has the lowest acoustic impedance, while the bone has the highest acoustic impedance [4]. The reflected signal received by the ultrasound probe also depends on the angle of incidence between the ultrasound beam and the tissue. If the incidence is 90 degrees, almost all the signals will be reflected back to the transducer. If the incidence is less than 90 degrees, only partial or no signals will be received by the probe. This is particularly important when we use ultrasound to guide the needle. During the ultrasound-guided out-plane approach and procedures, the needle is inserted perpendicular to the transducer. The needle shaft is imaged in a cross-sectional plane as a bright dot on the image. The tip of the needle position may not be reliably ascertained.

Some of the ultrasound properties directly affect the image quality. Frequency refers to the number of cycles of compressions and rarefactions in a sound wave. A higher frequency results in better resolution but limited penetration [5]. During ultrasound-guided procedures, it is better to choose a higher frequency for superficial structures, while a lower frequency for deeper structures. Attenuation is another property of ultrasound when it travels through tissues. A longer distance of travel and higher frequency waves result in greater attenuation. Increasing the gain in deeper areas of the interest can partially compensate.

Practice basic techniques

In order to perform successful ultrasound-guided nerve blocks, the providers have to master two basic skills: needle-to-ultrasound beam alignment and accurate positioning of the needle tip [6]. A basic comprehension of anatomy and its variations are only the groundwork of ultrasound-guided nerve blocks. Good hand-eye coordination is an essential skill for safely needle placement [7]. Visualization of the moving needle is important to perform a rapid and uneventful nerve block. An extensive practice is needed to learn to align the needle and ultrasound beam precisely [8].

The most common mistakes of novice users of ultrasound-guided nerve block are related to the view of the needle [9]. Failure to visualize the needle before advancement, frequent unintentional probe movement are the main reasons.

The needle guidance device has been designed to allow a better visualization of the needle. A study showed that the use of the needle guide device improved needle visualization, reduced procedure time, and increased user satisfaction scores [10]. However, the needle guide device used in the in-plane approach allowed the providers to see the needle 52% of the time [11]. In addition, the use of a needle guidance device prevents free transducer movement [12].

Training with phantoms is the first recommended step of training for ultrasound-guided nerve block [13]. The training models included gelatine-based phantoms (home-made and commercially available), water baths, tofu, and the use of a number of different animal tissues. Phantom selection has been determined by the cost of the phantom, tactile feedback, availability, and the skills to be gained [14]. A water bath and tofu are cheap, easy to obtain, and good for practising needle placement with no tactile feedback. Commercially available phantoms provide a firm texture that provides a degree of tactile feedback but are expensive and not available for many users.

A meat phantom (pork loin, chicken breast-based, and a roll of polony made from very finely minced meat) is cheap and provides tactile feedback close to human tissue. The use of animal tissue has a safety issue because the ultrasound machine can be contaminated by the bacteria in raw animal tissue. In addition, the meat phantoms are very time-consuming to construct.

In the operating rooms, many used or discarded items can be used as phantoms. A gelatin-based head doughnut, knee pad, and chest pad can be used for practice. There is no concern about contamination or hospital administration permission. The needle can be used repeatedly to minimize the cost.

A study of the learning curve showed inexperienced ultrasound users can improve their hand-eye coordination after practice on a gelatine phantom [7]. A study demonstrated that inexperienced users who have minimal instruction and no feedback can gain significant procedure skill [15]. However, participating in a training course or practice with experts can enhance the outcomes. Studies indicated that structured feedback during training for basic ultrasound skills improved the performance, enhanced the acquisition of skills, and augmented the effectiveness [16].

Common procedures for ultrasound-guided nerve blocks

The most practical and useful peripheral nerve blocks for upper extremity procedures are the supraclavicular and interscalene brachial plexus blocks. A femoral nerve/adductor canal block, in conjunction with a popliteal sciatic nerve block, is frequently utilized for lower extremity procedures. For abdominal procedures, the transversus abdominis plane block (TAP) is effective, relatively easy to learn, and rarely associated with severe complications. Indications, technical difficulty, success rate, and the complications of common procedures for ultrasound-guided nerve blocks are shown in Table 1.

**Table 1:** Indications, technical difficulty, success rate, and complications of common procedures for ultrasound-guided nerve blocks

Common peripheral nerve blocks	Indications	Ease to perform under ultrasound	Complete block success rate	Risk of complications
Supraclavicular Brachial Plexus Block	Upper extremity procedure distal to mid-humerus	Easy	High	Intermediate
Interscalene Brachial Plexus Block	Upper extremity procedure proximal to mid-humerus	Easy	High	High
Femoral Nerve Block	Anterior thigh procedures as well as medial lower extremity	Easy	High	Intermediate
Adductor Canal Block	Anterior thigh procedures as well as medial lower extremity	Easy	Intermediate	Low
Popliteal/Distal Sciatic Nerve Block	Distal lower extremity procedures, commonly encountered are Knee/Foot/Ankle procedures.	Intermediate	High	Intermediate
Subcostal Transversus Abdominis Plane Block	Incisional/somatic abdominal pain control above umbilicus	Easy	Intermediate	Intermediate
Classic Transversus Abdominis Plane Block	Incisional/somatic abdominal pain control below umbilicus	Intermediate	Intermediate	Low

Distal upper extremity block: supraclavicular brachial plexus block

Indications

Any upper extremity procedure distal to the mid-humerus; commonly encountered are the forearm and hand procedures.

Clinical Pearls

1. Patient in the supine position with the head up at approximately 30 degrees and turned to the contralateral side.

2. Put the linear probe along the clavicle as lateral as possible and then scan medially; the first artery encountered is the subclavian artery. The brachial plexus in the supraclavicular region sits lateral to the artery and superficial to the first rib (a bright/echogenic bone line with a shadow below) and the pleural (bright/echogenic line that moves with respiration).

3. Measure the distance from the skin to the brachial plexus and use this as the distance from the probe to the needle entry site in the skin. The needle takes a 45-degree angle and should be directed toward the “corner pocket” (between the first rib and the subclavian artery, where divisions from the C8 and T1 are located).

4. The first injection is at the corner pocket and local anesthetics should push the brachial plexus up; then the injection is given on top of the plexus.

Pitfalls/Troubleshooting

1. A failed block with ulnar nerve-sparing: this is almost always due to “corner pocket” failure when divisions from C8/T1 are not properly blocked. This can be prevented by the injection of a local anesthetic above the first rib and lateral to the subclavian artery.

2. Risk of pneumothorax in the supraclavicular block: this is high with the blind technique but has been significantly decreased with ultrasound guidance.

Proximal upper extremity block: interscalene block

Indications

Any upper extremity procedure proximal to the mid-humerus; commonly encountered are the shoulder and proximal humerus procedures.

Clinical Pearls

1. Position the patient and identify the brachial plexus, as described above in the supraclavicular block. Move the probe cephalad until the visualization of the classic traffic light sign of three nerve roots between two muscles: anterior scalene muscle medially and middle scalene muscle laterally.

2. Inject local anesthetics lateral to the nerve roots and medial to the middle scalene muscle, which is sufficient for a successful block.

Pitfalls/Troubleshooting

1. The highest risk of nerve injury is in all peripheral nerve blocks; therefore, efforts should be made to avoid performing this block under general anesthesia and there is no need to inject into or between the nerve roots.

2. Avoid injecting local anesthetics medical to nerve roots where the phrenic nerve is located

Anterior lower extremity block: femoral nerve/adductor canal block

Indications

A femoral nerve block (FNB) is indicated for anterior thigh procedures as well as the medial side of the entire lower extremity. An adductor canal block (ACB) is used to block the sensory branch of the femoral nerve (i.e. saphenous nerve) with or without the nerve to the vastus medialis at the adductor canal location, which offers the potential of less quadriceps femoris weakness than and comparable analgesic effects as a femoral nerve blockade.

Clinical Pearls

1. Place the patient supine with the lower extremity laterally rotated. For an FNB, place the linear probe in the inguinal crease to visualize the short axis view of the femoral artery. For ACB, place the linear probe in the middle one-third of the thigh to visualize the superficial femoral artery. The target nerve sits laterally to the artery.

2. Inject local anesthetics around the target nerve once the needle tip passes the fascia lata (for the FNB) or in the fascial layer between the sartorius and adductor longus/Magnus (ACB).

Pitfalls/Troubleshooting

1. A true FNB is performed at the level of the femoral artery, not after it bifurcates.

2. ACB can be performed anywhere along the length of the adductor canal, from the apex of the femoral triangle to the adductor hiatus.

Posterior lower extremity block (distal): popliteal/distal sciatic nerve block

Indications

The distal lower extremity procedures commonly encountered are the knee/foot/ankle procedures.

Clinical Pearls

1. This block can be placed with the patient in the supine, lateral, or prone position. Place the linear probe in the popliteal fossa to identify the popliteal artery, the vein, and the tibial nerve (and the bright/echogenic honeycomb structure superficial to the popliteal vein).

2. Put the tibial nerve in the center of the screen and watch it meet the common peroneal nerve coming superficially and laterally to form the sciatic nerve as the probe moves cephalad along the long axis of the thigh.

3. For better needle visualization, the distance between the skin and the sciatic nerve should be the same as that between the probe and the needle entry site on the skin. Maintain the needle trajectory parallel to the probe surface. For a faster onset, it is best to have local anesthetics around the nerve or the so-called “donut” sign.

Pitfalls/Troubleshooting

1. Always start injecting from the bottom of the nerve, therefore, pushing the nerve more superficially to improve resolution.

2. Pay particular attention to the anatomical relationship in the popliteal fossa, from superficial to deep in the order of the sciatic nerve-popliteal vein-popliteal artery. The popliteal vein is frequently not well visualized due to obesity and the pressure of the ultrasound probe. This can account for the negative aspiration yet intravenous injection of local anesthetics during this block performance. To minimize local anesthetic systemic toxicity, try to perform hydro-dissection when injecting at the bottom of the nerve.

Abdominal block: transversus abdominis Plane (TAP) block

Indications

For incisional/somatic abdominal pain control above the umbilicus, choose a subcostal TAP, whereas for pain below the umbilicus, use the classic TAP. These two compartments do not typically communicate, therefore, both subcostal and classic TAPs need to be performed for larger incisions such as these, extending from the xiphoid to the pubic symphysis.

Clinical Pearls

1. Subcostal TAP: The patient is in the supine position with a linear probe placed at the mid-clavicle line right below the rib case, local anesthetics are typically injected into the fascial layer between the rectus muscle and the transversus abdominis muscle.

2. Classic TAP: The patient is supine with the linear probe placed at the midaxillary line between the rib cage and iliac crest. Local anesthetics are typically injected into the fascial layer between the internal oblique and transversus abdominis muscles.

Pitfalls/Troubleshooting

1. Hydro-dissection with a small volume of saline to identify the correct fascia layer is a good way to save local anesthetics.

2. When finding it difficult to identify abdominal muscle layers, one can trace medically to the rectus muscle, which is always in continuation with the internal oblique, which tends to be the thickest among the external oblique and transversus abdominis muscles.

3. A TAP block offers excellent somatic pain control and has little effect on visceral pain.

Common procedures for ultrasound-guided vascular cannulation

An ultrasound can be used to guide vascular cannulation such as the central line, arterial line, and peripheral venous access.

Clinical Pearls

The high-frequency linear probe is often the probe of choice. Vascular structures can be visualized with the short-axis (SAX), long-axis (LAX) or a combination of two 【the oblique-axis (45 degree) 】. The SAX view offers better visualization of surrounding structures. The LAX view gives a better visualization of the needle during needle advancement. The SAX view is often combined with the out-of-plane approach, while the LAX and oblique axis are often combined with the in-plane technique [17]. The needle and the ultrasound beam are in the same plane with the in-plane technique. The needle is inserted perpendicular to the transducer and enters the ultrasound beam plane as a cross-sectional bright dot with the out-of-plane technique.

The SAX out-of-plane approach is easier to learn for the inexperienced user. However, this approach cannot visualize the entire needle or the depth of the needle. The LAX in-plane approach can show the depth of the needle and the entire needle. The oblique-axis is often combined with the in-plane technique [18]. It allows better visualization of the surrounding structures and needle advancement at the same time [19]. The peripheral venous and arterial access is mostly done with the SAX out-of-plane technique due to its superficial location and less need for appreciation of the needle depth. The central venous access is done by both the SAX out-of-plane and the LAX or oblique-axis in-plane technique depending on the performer’s experience.

Several techniques can be used to distinguish the arterial from venous vessels such as the shape of the vessel, color flow Doppler, and collapsible test. The artery is usually round and has a smaller diameter. The color flow Doppler can demonstrate the pulsatile blood flow in an artery. Normally, a vein is easily collapsible under pressure. Accidental artery puncture or failure to puncture the correct vessel usually results from a lack of the control of the needle depth and direction. The correct orientation of the probe, ensuring that the structure under the left aspect of the probe appears on the left side of the screen, may reduce the incidence of inappropriate directions.

## Conclusions

Ultrasound-guided techniques are very important tools for anesthesia providers. Inexperienced users can teach themselves to gain significant procedural skill. They have to master two basic skills: needle-to-ultrasound beam alignment and the accurate positioning of the needle tip by practice. Participation in training courses or practice with experts can enhance the outcomes. This article reviewed the indications, clinical pearls, and pitfalls of each procedure to help inexperienced users avoid potential complications.

## References

[1] La Grange P, Foster PA, Pretorius LK (1978). Application of the Doppler Ultrasound Blood Flow Detector in Supraclavicular Brachial Plexus Block. Br J Anaesth.

[2] Gray AT (2006). Ultrasound-guided regional anesthesia. Current state of the art. Anesthesiology.

[3] Marhofer P, Greher M, Kapral S (2004). Ultrasound guidance in regional anesthesia. Br J Anaesth.

[4] Kossoff G (2000). Basic physics and imaging characteristics of ultrasound. World J Surg.

[5] Lawrence JP (2007). Physics and instrumentation of ultrasound. Crit Care Med.

[6] Sites BD, Chan VW, Neal JM (2010). The American Society of Regional Anesthesia and Pain Medicine and the European Society of Regional Anaesthesia and Pain Therapy joint committee recommendations for education and training in ultrasound-guided regional anesthesia. Reg Anesth Pain Med.

[7] Kim S, Hauser S, Staniek A, Weber S (2014). Learning curve of medical students in ultrasound-guided simulated nerve block. J Anesth.

[8] de Oliveira Filho GR, Helayel PE, da Conceição DB, Garzel IS, Pavei P, Ceccon MS (2008). Learning curves and mathematical models for interventional ultrasound basic skills. Anesth Analg.

[9] Sites BD, Spence BC, Gallagher JD, Wiley CW, Bertrand ML, Blike GT (2007). Characterizing novice behavior associated with learning ultrasound-guided peripheral regional anesthesia. Reg Anesth Pain Med.

[10] Whittaker Whittaker, S. S., Lethbridge G, Kim C, Cohen ZK, Ng I (2013). An ultrasound needle insertion guide in a porcine phantom model. Anaesthesia.

[11] Tielens LK, Damen RB, Lerou JG, Scheffer GJ, Bruhn J (2014). Ultrasound guided needle handling using a guidance positioning system in a phantom. Anaesthesia.

[12] Van Geffen GJ, Mulder J, Gielen M, van Egmond J, Scheffer GJ, Bruhn J (2008). A needle guidance device compared to free hand technique in an ultrasound-guided interventional task using a phantom. Anaesthesia.

[13] Sujatta S (2015). First of all: do not harm! Use of simulation for the training of regional anaesthesia techniques: which skills can be trained without the patient as substitute for a mannequin. Best Pract Res Clin Anaesthesiol.

[14] Kwon SY, Hong SH, Park HJ, You Y, Kim YH (2015). The efficacy of lumbosacral spine phantom to improve resident proficiency in performing ultrasound-guided spinal procedure. Pain Med.

[15] Giglioli S, Boet S, De Gaudio AR, Linden M, Schaeffer R, Bould MD, Diemunsch P (2012). Self-directed deliberate practice with virtual fiberoptic intubation improves initial skills for anesthesia residents. Minerva Anestesiol.

[16] de Oliveira Filho GR, Mettrau FAC (2018). The effect of high-frequency, structured expert feedback on the learning curves of basic interventional ultrasound skills applied to regional anesthesia. Anesth Analg.

[17] Saugel B, Scheeren TWL, Teboul JL (2017). Ultrasound-guided central venous catheter placement: a structured review and recommendations for clinical practice. Critical Care.

[18] Dietrich CF, Horn R, Morf S (2016). Ultrasound-guided central vascular interventions, comments on the European Federation of Societies for Ultrasound in Medicine and Biology guidelines on interventional ultrasound. J Thorac Dis.

[19] Wilson JG, Berona KM, Stein JC, Wang R (2014). Oblique-axis vs. short-axis view in ultrasound-guided central venous catheterization. J Emerg Med.

